# Research status and global trends of late-life depression from 2004 to 2023: bibliometric analysis

**DOI:** 10.3389/fnagi.2024.1393110

**Published:** 2024-04-30

**Authors:** Ruonan Du, Kebing Yang, Wei Li, Zhiren Wang, Haipeng Cai

**Affiliations:** Huilongguan Clinical Medical School of Peking University, Beijing Huilongguan Hospital, Beijing, China

**Keywords:** late-life depression, dementia, cognitive impairment, CiteSpace, bibliometric analysis, global trends

## Abstract

**Background:**

Global research hotspots and future research trends in the neurobiological mechanisms of late-life depression (LLD) as well as its diagnosis and treatment are not yet clear.

**Objectives:**

This study profiled the current state of global research on LLD and predicted future research trends in the field.

**Methods:**

Literature with the subject term LLD was retrieved from the Web of Science Core Collection, and CiteSpace software was used to perform econometric and co-occurrence analyses. The results were visualized using CiteSpace, VOSviewer, and other software packages.

**Results:**

In total, 10,570 publications were included in the analysis. Publications on LLD have shown an increasing trend since 2004. The United States and the University of California had the highest number of publications, followed consecutively by China and England, making these countries and institutions the most influential in the field. Reynolds, Charles F. was the author with the most publications. The *International Journal of Geriatric Psychiatry* was the journal with the most articles and citations. According to the co-occurrence analysis and keyword/citation burst analysis, cognitive impairment, brain network dysfunction, vascular disease, and treatment of LLD were research hotspots.

**Conclusion:**

Late-life depression has attracted increasing attention from researchers, with the number of publications increasing annually. However, many questions remain unaddressed in this field, such as the relationship between LLD and cognitive impairment and dementia, or the impact of vascular factors and brain network dysfunction on LLD. Additionally, the treatment of patients with LLD is currently a clinical challenge. The results of this study will help researchers find suitable research partners and journals, as well as predict future hotspots.

## Introduction

Late-life depression (LLD), one of the most common psychiatric disorders in the elderly population, refers to depressive disorders in adults ≥ 60 years of age (Alexopoulos, 2005; Taylor, 2014), which can be categorized into early-onset depression and late-onset depression. The former refers to depressive disorders with the first onset before age 60 and recurrence after age 60, whereas the latter refers to depressive disorders with the first episode after age 60 (Ly et al., 2021). With global population aging, the prevalence of LLD is increasing every year (Jellinger, 2023). A recent report showed that the prevalence of depressive symptoms in older adults is 20% and is highly heterogeneous (Tang et al., 2022). Other reports have shown that LLD affects 1.8 to 7.2% of older adults in the community (Beekman et al., 1999; Blazer, 2003) and that between 10 and 15 percent of older adults have significant depressive symptoms even without a diagnosis of depressive disorder (Kok and Reynolds, 2017). LLD is associated with a variety of adverse outcomes, such as reduced quality of life, negative impact on somatic comorbidities, and increased suicidal and non-suicidal mortality. LLD is a main cause of high medical co-morbidity and mortality in older adults (Valiengo Lda et al., 2016). It was shown that the risk of death in older adults with depressive disorders is 1.65 times higher than in non-depressed older adults. Overall, LLD and its associated medical comorbidities have a serious impact on healthcare utilization and costs (Unützer et al., 2009), and place a significant financial burden on caregivers and society.

Late-life depression is a heterogeneous disorder that varies in presentation among patients. Although LLD has the core features of depressive disorders, symptoms of anxiety, agitation, and somatic symptoms (e.g., headaches and muscle aches) are more likely to present in LLD than in depressive disorders in other age groups (Fiske et al., 2009). In addition, patients with LLD often demonstrate more pronounced neurocognitive symptoms such as memory loss and impaired executive function. These cognitive symptoms may not resolve fully after antidepressant treatment. Studies have shown that LLD increases the risk of all-cause dementia, particularly vascular dementia (VD) and Alzheimer’s disease (AD) (Diniz et al., 2013), leading to an increasing number of researchers becoming interested in the association between LLD and dementia.

Currently, the treatment for LLD is based on medication and psychotherapy (Gundersen and Bensadon, 2023). Due to the specificities of the elderly population (e.g., comorbid somatic conditions or cognitive impairment), the diagnosis and treatment of depressive disorders tend to be more difficult than in younger people. Treatment of LLD has been found to be inadequate; as patients’ cognitive function declines and somatic conditions worsen, they turn to antidepressant treatment. However, the duration of antidepressant treatment is usually too short, or the dose of antidepressant medication is lower than the recommended dosage (Barry et al., 2012).

Elderly patients have a greater burden of physical illnesses such as hypertension, diabetes, and cardiovascular disease; therefore, antidepressants may be less effective in older patients. A meta-analysis of patients aged > 60 years showed that the efficacy of antidepressant medications declines with age (Calati et al., 2013). For the treatment of patients with LLD, clinicians must consider both the possibility of drug–drug interactions and the effects of medications on cognitive functioning, which adds to the complexity of antidepressant treatment for patients with LLD. In addition, non-pharmacological treatments, such as psychotherapy (e.g., cognitive and interpersonal therapy) or exercise therapy, may be helpful in the rehabilitation of patients with LLD; however, evidence in this area is limited.

Currently, there is increasing interest in the neurobiological mechanisms of LLD as well as its diagnosis and treatment; however, global research hotspots and future research trends in this field are not yet clear. Bibliometric methods allow quantitative analysis of publications, visualization of scientific and technical knowledge, and internal connections (Hicks et al., 2015). Analyzing elements such as authors, countries, keywords, and citations reveals research content, current research hotspots, and future research trends in a particular field (Ninkov et al., 2022). This study aims to clarify the global trends in research on LLD, further understand the research hotspots in the field, and predict future research trends through bibliometric analysis.

## Materials and methods

### Data sources

**FIGURE 1:**
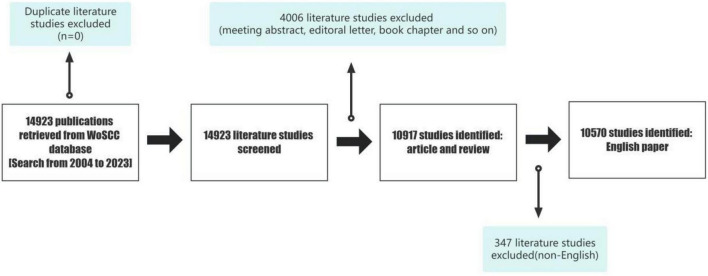
Flow chart of the study.

The data for this study were retrieved from the Web of Science Core Dataset (WoSCC), which includes several datasets and is one of the most commonly used databases for bibliometric analyses. We retrieved data from the WoSCC database on 30 December 2023 (Figure 1).

Search strategy: [TI = (elderly OR geriatric OR “late-life” OR “late onset” OR older OR “old age”) AND TI = (depress* OR major depressive disorder)] OR [AK = (elderly OR geriatric OR “late-life” OR “late onset” OR older OR “old age”) AND AK = (depress* OR major depressive disorder)].

Document type: Article and Review.

Language: English.

A total of 14,923 papers on LLD were retrieved using these keywords. After restricting document type and language, the remaining 10,570 papers comprised mostly original articles and reviews.

### Introduction of bibliometric instruments

CiteSpace is a Java program developed by Prof. Chao-Mei Chen of Drexel University for visualizing databases. CiteSpace incorporates methods such as cluster analysis, social network analysis, and multidimensional scale analysis, focusing on detecting and analyzing the evolutionary trends of disciplinary research fronts, the relationship between the research fronts and their knowledge bases, and the internal linkages between different research fronts (Chen, 2006). Core countries/regions, institutions, authors, and their partnerships can be identified through collaborative networks. Co-occurring keywords can reveal the basis and hotspots of research. In addition, the detection of emergence can be more intuitive in identifying trends in a certain field, providing scholars with scientific and intuitive references and auxiliary support. For data analysis, we used VOSviewer, an econometric analysis software developed by Nees Janvan Eck and Ludo Waltman, Leiden University, to construct and visualize knowledge network maps (van Eck and Waltman, 2010). Based on the principle of co-citation, it has strong visualization capabilities and is suitable for large-scale sample data.

### Data analysis and visualization

The analyses in this study used the WoSCC to analyze data, such as annual publication volume, year of publication, and h-index. The h-index is one of the most commonly used indices for evaluating scholars’ academic influence. It is a comprehensive assessment method with both quantitative and qualitative evaluations, which can reflect scholars’ academic level and academic influence more objectively; a high h-index indicates that scholars have great influence.

Keywords were used to search for relevant literature and filter ineligible literature, and the selected literature information was saved in.txt format. The data were imported into CiteSpace v6.2.R6, and duplicate literature was eliminated. Nodes were selected according to the type of analysis to be performed, with the following parameter settings: time slice: January 2004–December 2023; terminology sources: title, abstract, author keyword, and keyword; node types: author, institution, country, keyword, citation, citing author, and citing journal; and selection criterion: Top *N* = 50. CiteSpace was used for co-occurrence analysis and visualization of keywords and cited literature. Additionally, we used VOSviewer to analyze the country/region or institution distribution and keyword co-occurrence of relevant studies and visualized the results using the Bibliometrix R package (Aria et al., 2021). The number of annual publications was visualized and predicted using Microsoft Excel 365.

In the visualization images generated by the software, the larger the node, the higher the keyword co-occurrence frequency. The centrality of a node refers to the number of shortest paths passing through the node in the network, and is a measure of the size of the node’s connectivity role in the overall network (Yiyang and Jingjing, 2015). The higher the centrality of a node, the more times it appears on the shortest paths in the overall network and the greater its influence and importance.

## Results

### Distribution of annual publication

**FIGURE 2:**
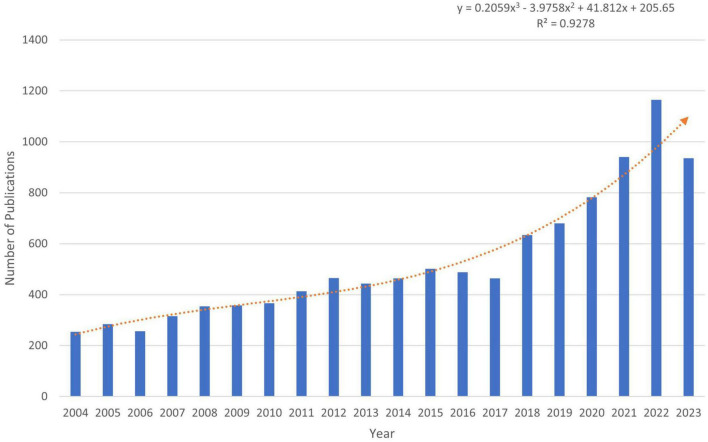
The number of publications from 2004 to 2023.

Changes in the number of papers are important indicators of trends in the field. Since 2004, the number of publications with LLD subject headings has been increasing annually. As of 18 December 2023, 10,570 articles and reviews have been published, indicating that LLD is attracting increasing attention from researchers. We predicted the number of publications using Excel, where the predicted growth model equation was *y* = 0.2059x^3^ − 3.9758x^2^ + 41.812x + 205.65, with *R*^2^ = 0.928, x representing (forecast year minus 2004), and y representing the predicted number of publications per year (Figure 2). According to the prediction formula, more than 1,310 publications on related topics are expected to be published by 2025, and this field is expected to attract the attention of an increasing number of researchers.

### Analysis of scientific collaboration network

**FIGURE 3:**
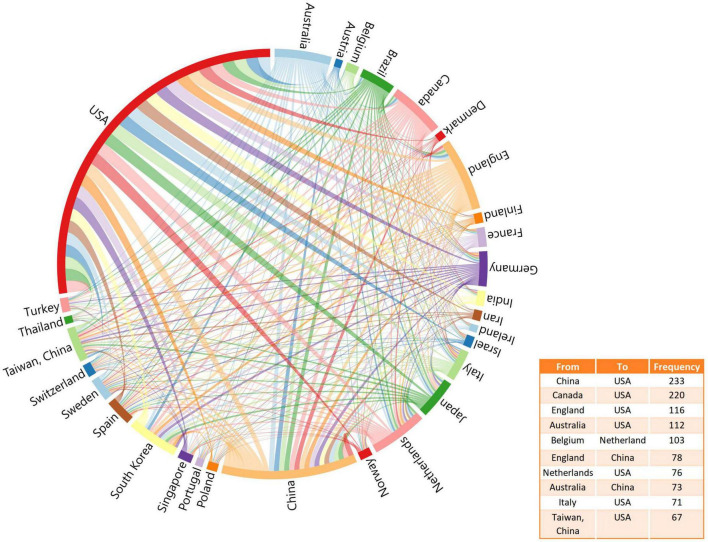
Collaborative research between countries on LLD according to frequency.

A total of 116 countries have published articles on LLD. The United States had the highest number of publications in this area (*n* = 3,940; 37.18%), followed by China (*n* = 1,501; 17.78%) and England (*n* = 810; 7.64%), as shown in Table 1. Figure 3 shows the network of collaborations between countries, with the top ten inter-country/inter-region collaborations. Cooperation between China and the United States was the most common, followed by the United States and Canada, and the United States and England.

**TABLE 1:** The top 10 productive countries/regions of the topic.

Country	Number of publications	Percentage (%)
USA	3,940	37.18
China	1,501	14.20
England	810	7.64
Australia	625	5.90
Netherlands	615	5.80
Canada	600	5.67
South Korea	562	5.30
Japan	436	4.11
Germany	384	3.62
Taiwan, China	383	3.58

**FIGURE 4:**
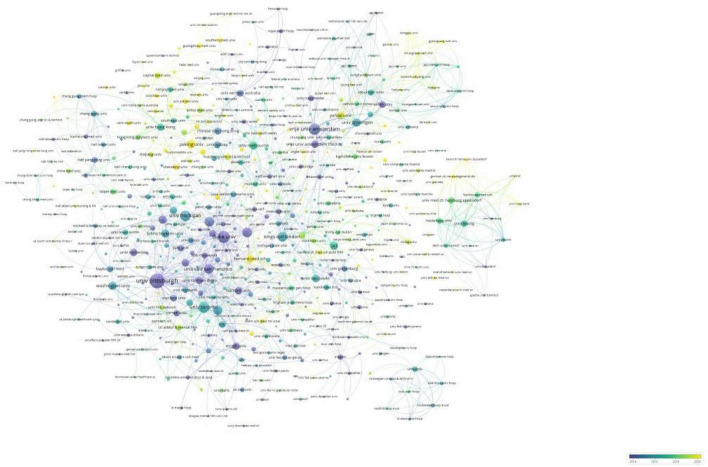
Overlay visualization of institution for LLD research.

Table 2 lists the top ten institutions in terms of the number of publications they produced, as well as the centrality of each institution. The top three institutions in terms of relevant publications were the University of California, Pennsylvania Commonwealth System of Higher Education, and the University of Pittsburgh. The centrality of a node can be used to measure its influence and connectivity in the network. The top three institutions in terms of centrality were Duke University, Vrije University Amsterdam, and the University of Pittsburgh, which suggests that they had more extensive collaborations with other institutions. We also analyzed the collaborative network and global distribution of institutions. Figure 4 shows the evolution of the issuing institutions in the field over the last 20 years. Nodes colored toward yellow indicate a higher number of recent publications and more recent generation of scholarly impact, while those colored toward purple represent a higher number of articles published in the early years. Figure 4 shows several academic institutions that recently gained high influence: Peking University, Harvard Medical School, Shandong University, and Southern Medical University. Interestingly, most of these institutions are located in China, demonstrating the gradual expansion of China’s influence in this field in recent years.

**TABLE 2:** Top 10 institutions according to publications and the corresponding centrality.

Rank	Name	Number	Centrality
1	University of California	497	0.01
2	Pennsylvania Commonwealth System of Higher Education (PCSHE)	476	0.01
3	University of Pittsburgh	409	0.03
4	Vrije University Amsterdam	382	0.04
5	US Department of Veterans Affairs	367	0.01
6	University of London	363	0.02
7	Duke University	277	0.08
8	Harvard University	249	0.01
9	University of Toronto	239	0.03
10	University of Texas System	216	0.03

Table 3 lists the top ten authors according to the number of publications, as well as their institutions and h-index. The author with the most publications is Reynolds, Charles F., whose h-index is among the top three most influential authors.

**TABLE 3:** Top 10 authors according to publications, the corresponding institution and h-index.

Rank	Author	Publications	Institution	h-index
1	Reynolds, Charles F.	208	University of Pittsburgh	71
2	Steffens, David C.	152	University of Connecticut	72
3	Mulsant, Benoit H.	131	University of Toronto	80
4	Alexopoulos, George S.	112	Weill Cornell Medicine	70
5	Comijs, Hannie C.	111	Vrije Universiteit Amsterdam	53
6	Beekman, Aartjan T. F.	103	Vrije Universiteit Amsterdam	43
7	Eric J Lenze	86	Washington University	51
8	Butters, Meryl A.	81	University of Pittsburgh	60
9	Voshaar, R. C. Oude	80	University of Groningen	41
10	Karp, Jordan F.	73	University of Arizona	31

### Analysis of journals, co-cited journals and co-cited references

According to the WoSCC database, articles on LLD have been published in 1,630 different journals. The top ten journals and co-cited journals mainly cover the fields of geriatrics, neuroscience, and clinical psychology (Table 4), and most of the journals are in the Q1 region of the Journal Citation Reports (JCR) quartile and have a high impact factor, which suggests that the quality of articles in the field of LLD is high. The journal with the highest number of publications is *International Journal of Geriatric Psychiatry*, followed by *American Journal of Geriatric Psychiatry*, and *Journal of Affective Disorders*, and four of the top ten published journals have an impact factor higher than 6.00.

**TABLE 4:** Top 10 journals and co-cited journals.

Items	Ranking	Name	Counts	IF2023	JCR
Journal	1	*International Journal of Geriatric Psychiatry*	615	4.00	Q2
	2	*American Journal of Geriatric Psychiatry*	564	7.22	Q1
	3	*Journal of Affective Disorders*	545	6.60	Q1
	4	*Aging Mental Health*	467	3.40	Q1
	5	*International Psychogeriatrics*	287	7.00	Q1
	6	*Archives of Gerontology and Geriatrics*	185	4.00	Q2
	7	*BMC Geriatrics*	155	4.1	Q2
	8	*Journal of the American Geriatrics Society*	147	6.30	Q1
	9	*Frontiers in Psychiatry*	116	4.70	Q2
	10	*Psychogeriatrics*	108	2.00	Q4
Co-cited journals	1	*International Journal of Geriatric Psychiatry*	5,242	4.00	Q2
	2	*Journal of Affective Disorders*	4,998	6.60	Q1
	3	*American Journal of Geriatric Psychiatry*	4,691	7.22	Q1
	4	*American Journal of Psychiatry*	4,293	17.7	Q1
	5	*Journal of the American Geriatrics Society*	4,269	6.30	Q1
	6	*Archives of Gerontology and Geriatrics*	4,035	4.0	Q2
	7	*British Journal of Psychiatry*	3,705	10.5	Q1
	8	*Psychological Medicine*	3,443	6.90	Q1
	9	*JAMA*	3,149	120.70	Q1
	10	*International Psychogeriatrics*	3,080	7.00	Q1

An analysis of journal co-citations revealed the contribution of each journal to the field. The top three journals based on co-citation counts were *International Journal of Geriatric Psychiatry* (5,242), *Journal of Affective Disorder* (4,998), and *American Journal of Geriatric Psychiatry* (4,691). Table 5 shows information about the top ten highly cited articles published in journals in Q1. Three of these studies were published in *JAMA* and its subseries, and the most recent was a review of the management of LLD by Kok and Reynolds (2017).

**TABLE 5:** Top 10 highly cited references.

Rank	Times cited	References	Title (publication year)	Journal (IF2023)
1	1,284	Fiske et al., 2009	Depression in older adults (2009)	*Annual Review of Clinical Psychology* (18.4/Q1)
2	1,104	Alexopoulos, 2005	Depression in the elderly (2005)	*Lancet* (168.9/Q1)
3	770	Diniz et al., 2013	Late-life depression and risk of vascular dementia and Alzheimer’s disease: systematic review and meta-analysis of community-based cohort studies (2013)	*British Journal of Psychiatry* (10.5/Q1)
4	719	Bruce et al., 2004	Reducing suicidal ideation and depressive symptoms in depressed older primary care patients–a randomized controlled trial (2004)	*JAMA* (120.7/Q1)
5	714	Djernes, 2006	Prevalence and predictors of depression in populations of elderly: a review (2006)	*Acta Psychiatrica Scandinavica* (6.7/Q1)
6	674	Santini et al., 2020	Social disconnectedness, perceived isolation, and symptoms of depression and anxiety among older Americans (NSHAP): a longitudinal mediation analysis (2020)	*Lancet Public Health* (50.0/Q1)
7	457	Butters et al., 2004	The nature and determinants of neuropsychological functioning in late-life depression (2004)	*JAMA Psychiatry* (25.8/Q1)
8	390	Soysal et al., 2017	Relationship between depression and frailty in older adults: a systematic review and meta-analysis (2017)	*Aging Research Reviews* (13.1/Q1)
9	387	Kok and Reynolds, 2017	Management of depression in older adults a review (2017)	*JAMA* (120.7/Q1)
10	373	Bora et al., 2013	Cognitive impairment in euthymic major depressive disorder: a meta-analysis (2013)	*Psychological Medicine* (6.9/Q1)

### Changes in trends of research disciplines

**FIGURE 5:**
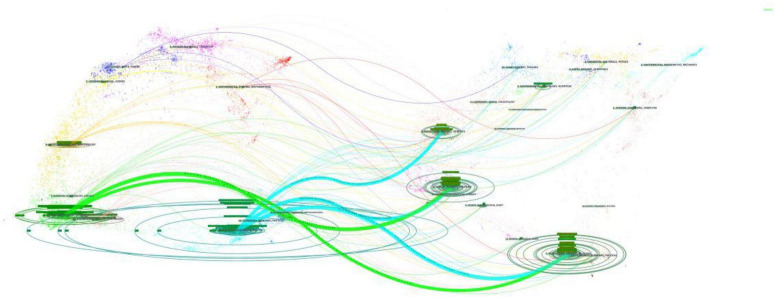
Dual-map overlay of article citations for LLD research.

The results of the disciplinary crossover were visualized using CiteSpace’s dual-map overlay analysis feature, and Figure 5 shows the citation relationships between journals. The left side of the image shows the citing journals, the right side shows the cited journals, and the three bold blue lines and two green lines are the core citation paths. Green citation paths represent articles published in MEDICINE/MEDICAL/CLINICAL-themed journals, which are usually cited by articles in HEALTH/NURSING/PSYCHOLOGY/EDUCATION/SOCIAL-themed journals. The blue citation paths indicate that articles in journals with PSYCHOLOGY/EDUCATION/HEALTH themes were mostly cited by articles in journals with MOLECULAR/BIOLOGY/GENETIC/NURSING/PSYCHOLOGY/ MEDICINE themes.

### Analysis of co-occurring keywords

**FIGURE 6:**
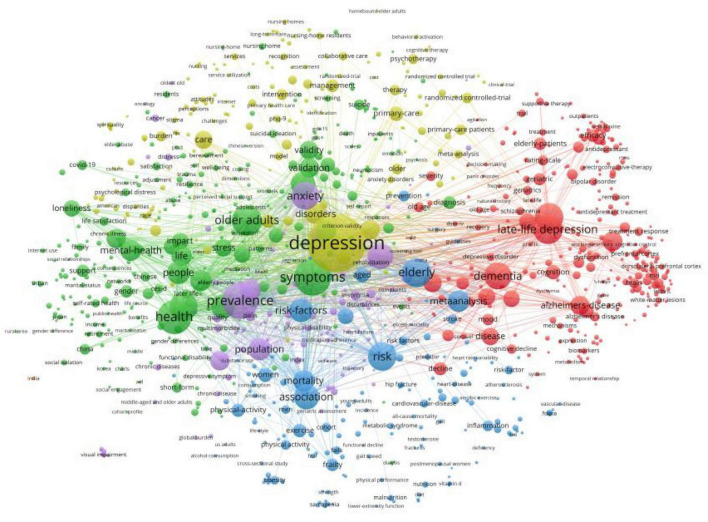
Co-occurrence network visualization of keywords in VOSviewer.

Table 6 lists the top ten keywords according to their frequency of occurrence. The most frequently occurring keyword was “late-life depression,” followed by “older adult,” “depressive symptoms,” “dementia,” and “prevalence.” We visualized the keyword co-occurrence results using VOSviewer (Figure 6), which shows the number of occurrences of the keywords < 15 times. Nodes of different colors represent keywords in different clusters, and the size of the nodes indicates their frequency of occurrence.

**TABLE 6:** Top 10 keywords in terms of frequency in the research.

Rank	Keyword	Frequency
1	Late-life depression	3,617
2	Older adult	3,562
3	Depressive symptoms	2,159
4	Dementia	2,056
5	Prevalence	1,968
6	Health	1,761
7	Major depression	1,315
8	Risk factor	1,246
9	Quality of life	932
10	Cognitive impairment	902

We clustered the co-occurring keywords to identify popular research topics. In general, the clustering results were considered reliable when the silhouette was > 0.7. We used the log-likelihood ratio (LLR) clustering method to obtain eight clustered tags (Table 7), of which seven tags had a silhouette value > 0.7. The clustered tag with the largest silhouette value was #7 (silhouette = 0.999), with the tag “depression in Alzheimer’s disease.” Based on the analysis of co-occurring keywords and clustering results, late-life depression, dementia, magnetic resonance imaging (MRI), and cardiovascular disease were popular research topics.

**TABLE 7:** Clusters of co-occurring keywords.

Cluster	Size	Silhouette	Label (LLR algorithm)	Most cited keywords in cluster
0	148	0.813	MRI	White matter; vascular depression; hippocampus
1	135	0.764	Depression	Epidemiology; prevalence; older adults
2	129	0.675	Antidepressant	Double blind; elderly patient; serotonin reuptake inhibitor
3	122	0.745	Collaborative care	Collaborative care; management; psychotherapy
4	93	0.766	Alzheimer’s disease	cognition; mild cognitive impairment; memory
5	84	0.739	Stress	Affective disorder; cognitive decline
6	66	0.772	Cardiovascular disease	BDNF; inflammation; biomarkers
7	4	0.999	Depression in Alzheimer’s disease	Arterial stiffness; brain disease model

### Analysis of burst keywords and citation

**FIGURE 7:**
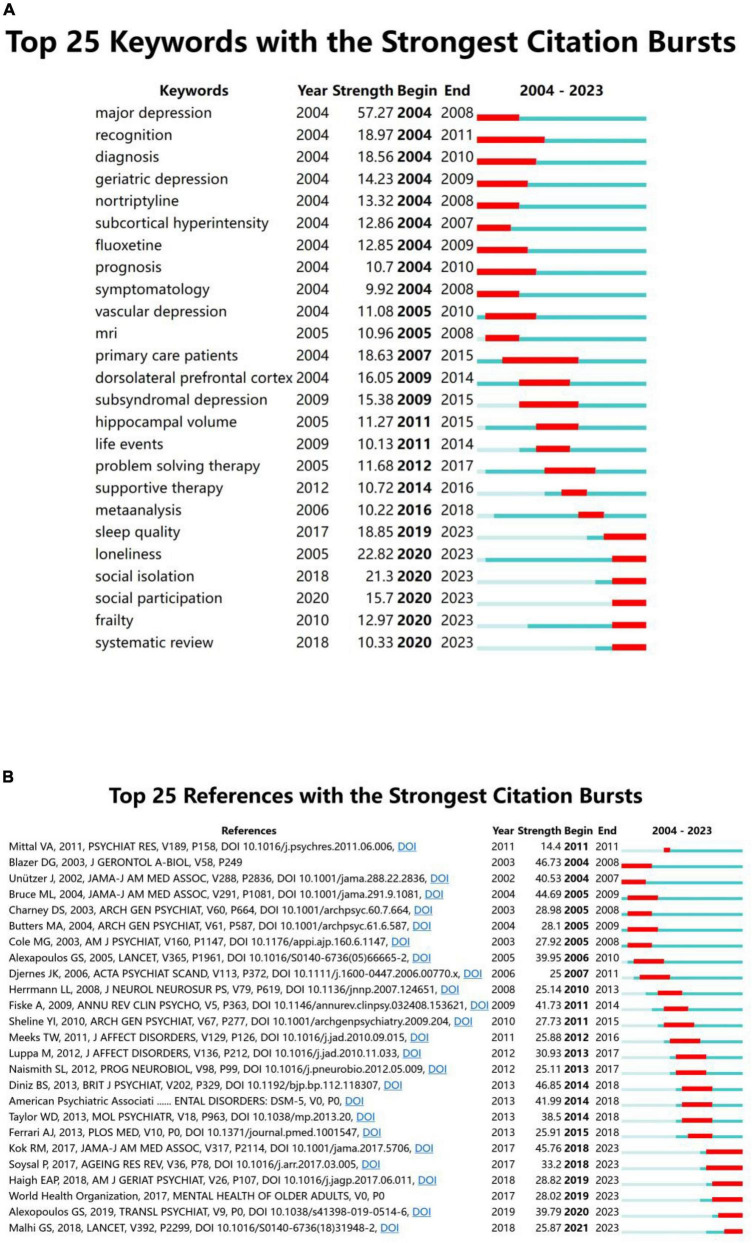
**(A)** Top 25 keywords with the strongest citation bursts. **(B)** Top 25 references with the strongest citation bursts.

Keyword bursts reflect hotspots and trends in the research field during a certain period. In Figure 7A, the blue line represents the period 2004–2023, and the red line represents the duration of keyword bursts. Figure 7A shows the 25 most-cited keywords. “Major depression” is the strongest keyword in the last 20 years (strength: 57.27), and the keywords that last until 2023 include “sleep quality,” “loneliness,” “social isolation,” “social participation,” “frailty,” and “systematic review,” reflecting the latest research trends.

Citation bursts in references illustrate the evolution of knowledge domains. Figure 7B lists the 25 references with high citation durations. The durations of the citation bursts are indicated by the red line segments. The strongest burst being an article published in *The British Journal of Psychiatry* in 2013 by Diniz et al. (2013). Their research team conducted a systematic review and meta-analysis of prospective studies with large samples of patients with LLD to assess the risk of dementia. Six reference bursts lasted until 2023 (Kok and Reynolds, 2017; Soysal et al., 2017; World Health Organization, 2017; Haigh et al., 2018; Malhi and Mann, 2018; Alexopoulos, 2019).

## Discussion

This study was the first to apply bibliometric analysis methods to investigate the topic of LLD. Relevant articles published between 2004 and 2023 were visualized and analyzed to detect global hotspots and trends. The findings of this study will help researchers identify the research hotspots in this field and choose appropriate research directions.

### Global trends in LLD

The number of publications in this field has increased since 2004. The United States and China take first and second place for number of publications, accounting for more than 50 percent of total publications and making a large contribution to the field. The University of California has the most publications in the field, followed by PCSHE. The author with the most publications is Reynolds, Charles F. from the University of Pittsburgh, whose high h-index indicates strong academic influence in the field. The ten countries with the most articles have published a total of 10,194 articles, contributing the vast majority of articles in the field (10,194/10,570). However, cooperation between countries or institutions needs to be strengthened, and our findings will help researchers find potential partnerships.

### Status of publications

Most of the articles in this field were published in journals related to geriatrics, neuroscience, and clinical psychology. The journals had high impact factors, with most distributed in the Q1 and Q2 zones, indicating that the quality of the research in this field is high, leading to increased interest. The journal with the highest number of articles and citations is *International Journal of Geriatric Psychiatry*, which provides an international perspective on geriatric psychiatry, publishing articles on topics such as the epidemiology of mental disorders in the elderly, clinical etiological studies, neurochemical studies, treatment trials, and geriatric psychiatric nursing. The majority of the ten most-cited articles dealt with LLD’s relationship with cognitive functioning and disease management, with five of them published in top journals such as *JAMA* and its sub-journals.

The most co-cited article was by Fiske et al. (2009), in which the authors describe the characteristics of LLD in terms of epidemiology, protective and risk factors, consequences, and treatments, summarizing some of the emerging approaches to prevention. Although the prevalence of depression appears to be lower in older adults than in younger adults, the serious consequences should not be ignored, with risk factors including genetic susceptibility, cognitive biases, age-related neurobiological changes, and stressful events. Most evidence suggests that pharmacological and psychological treatments are more effective in patients with LLD, which may be related to improvements in their overall physical functioning during treatment. However, LLD has a poorer prognosis, and its treatment is more challenging than that in young adults, with a higher incidence of comorbidities and adverse events due to the use of multiple medications (Piel and Quante, 2023); therefore, it is essential to individualize treatment regimens for different individuals. Additionally, the development of effective and low-cost preventive measures for the majority of older adults remains a major challenge in the prevention of LLD (Vyas and Okereke, 2020).

### Research focus on LLD

We predict the following future research hotspots by interpreting the cluster analysis of co-occurring results and bursts in terms of keywords and citation results.

#### LLD, cognitive impairment, and dementia

Cognitive impairment is one of the main features of LLD, and the two often coexist. Unlike the normal aging process, cognitive impairment in LLD patients may be associated with dementia. Cognitive impairment in patients with LLD has been shown to be more severe than that in early adult depressive disorders (Invernizzi et al., 2021), which may be both a reflection of organic brain lesions in old age and a predictor of an increased risk of dementia. Cognitive impairment in LLD is multidimensional, usually characterized by reduced executive functioning, impaired attention, and delayed memory extraction. Even when depressive symptoms improve, cognitive impairment may persist for longer periods. A study comparing the differences between cognitive impairment in depressive disorders at different times found that cognitive impairment before age 60 was mainly characterized by executive impairment syndrome (impairment of attention, psychomotor, or short-term memory functions) (Castaneda et al., 2008). In contrast, LLD has more severe impairments in verbal learning, memory, and motor speed, compared to younger depressive disorders (Thomas et al., 2009).

A growing number of researchers suggest that depression may represent the first symptom of a neurodegenerative process (Wright and Persad, 2007). Depression may increase the risk of cognitive decline in people with cognitive integrity and further impair cognitive functioning in people with mild cognitive impairment (MCI) more than those without depressive symptoms (Ismail et al., 2014). One study showed that the risk of developing dementia later in life with depressive symptoms in old age ranged from 1.16 to 3.50 (Jorm, 2000). Another study from China, which followed older adults with MCI for two years, found that patients with MCI who presented with depressive symptoms at baseline had an increased risk of developing dementia later in life (Chan et al., 2011). More importantly, of all the neuropsychiatric symptoms assessed at baseline (hallucinations, delusions, depression, anxiety, apathy, agitation, etc.), only depressive symptoms proved to be a risk factor for progression to dementia (Chan et al., 2011). This finding suggests the value of timely detection of possible depressive symptoms in older adults with cognitive impairment, which would facilitate early identification and intervention in high-risk populations.

In clinical practice, physicians often encounter patients with depressive symptoms combined with cognitive impairment; however, it is often difficult to determine the sequence of symptoms, which should highlight the possibility that depressive symptoms may be a risk factor for the emergence or aggravation of cognitive impairment. Detailed medical history should be obtained from the patient and their family to understand not only the evolution of the depression but also the pattern of change in cognitive function, to assess their current level of cognitive functions and ability to perform activities of daily living, and to improve neuroimaging examinations. In a recent study that analyzed voxel-based morphometry in patients in order to distinguish structural brain differences between LLD-related MCI and prodromal AD, they found that regional gray matter loss may reflect amyloid deposition and depression-induced damage, and that such changes may be helpful in distinguishing between LLD-related MCI and prodromal AD, leading to earlier intervention (Hyung et al., 2021). While treating depressive symptoms, doctors should regularly follow-up with patients to monitor for further cognitive decline and the risk of developing dementia at a later stage. This will enable doctors to implement timely interventions for patients with cognitive impairment. A large-sample prospective cohort study by Yang et al. explored the relationship between depression, its treatment, and the risk of developing dementia (Yang et al., 2023). The team recruited 354,313 patients with depressive disorders from UK Biobank and found that depression was associated with an increased risk of dementia. The longer the course of depression, the higher the risk of dementia (Yang et al., 2023). Another large-scale European follow-up study reached similar conclusions (Wu et al., 2020). In addition, untreated patients with depression had a 30 percent higher risk of developing dementia than those who received medication or psychotherapy (Yang et al., 2023). The results provide additional evidence for the need to treat LLD as early as possible.

Certain researchers consider depression to be a predictive risk factor for dementia (Saczynski et al., 2010; Wu et al., 2020), whereas others consider depression a prodromal symptom of dementia (Ganguli et al., 2006; Mirza et al., 2014; Singh-Manoux et al., 2017). More than 40 million people worldwide have been diagnosed with dementia, a number that is expected to continue to increase over the next 20 years (Scheltens et al., 2021). A previous meta-analysis reported that late-life depression is associated with dementia (Diniz et al., 2013). However, research on the relationship between the two is inconclusive (Becker et al., 2009). More long-term follow-up studies with larger sample sizes are needed to clarify this relationship.

#### Brain network dysfunctions

The clinical manifestations of LLD involve multiple psychiatric symptom dimensions and cognitive impairment domains. Several imaging studies have explored the structural and functional mechanisms of the brain in patients with LLD and the brain dysfunction associated with LLD. Researchers have proposed that there may be different clinical subtypes of LLD associated with disruptions in cognitive and affective functioning due to brain network dysfunction (Etkin et al., 2015; Samanez-Larkin and Knutson, 2015; Mather, 2016), often poorly responsive to available treatments.

The most widely studied brain networks in LLD are the executive control network, default mode network, and salience network, which are related to executive attention and regulatory processes; abnormalities in these processes may lead to negative affective experiences and cognitive symptoms in patients with LLD. Various etiological mechanisms may lead to brain network disorders, including vascular diseases and endocrine and immune system abnormalities (Direk et al., 2012; Taylor et al., 2013; Jaywant et al., 2022). The relationship between white matter lesions or hyperintensities (WMH) and brain functional networks in LLD has attracted scholarly attention. The findings of Seiler et al. (2018) suggest that WMH may lead to a decrease in white matter structural connectivity, which makes the overall efficiency of information exchange between brain regions impaired (Langen et al., 2018). A recent study using MRI data from a large elderly population sample found that larger WMH burdens are associated with decreased resting-state functional connectivity, and local white matter lesions may reduce the functional connectivity of specific fiber bundles (Langen et al., 2017). The severity of WMH is associated with the prevalence of LLD and antidepressant response rates (Gunning-Dixon et al., 2010; Park et al., 2015; van Sloten et al., 2015), while damage to white matter within the aforementioned brain networks may lead to abnormalities in functional networks, which can affect cognitive and emotional processing (Tully et al., 2017). A meta-analysis of the general population by Arnone et al. (2012) showed that white matter lesions in patients with major depressive disorder (MDD) worsened with increasing age. Cotter et al. (2020) followed 716 older adults with intact function and found a negative correlation between age and negative mood, which appeared to diminish or even reverse after age 71. The integrity of white matter microstructure, executive function, and processing speed seems to have a positive effect on the maintenance of a positive mood, suggesting that a deeper understanding of the patterns of abnormal networks may help discover new treatments.

Some patients with LLD are less responsive to existing treatment regimens; therefore, researchers have proposed the use of cognitive/behavioral assessments, MRI sequences, and computational methods to develop individualized interventions for disrupted brain network connections. A randomized controlled study by Murri et al. (2018) compared the efficacy of sertraline alone versus sertraline combined with physical activity in patients with LLD; the results suggested that antidepressant medication combined with physical activity is clinically advantageous, and physical activity may be a safe, effective, and cost-effective augmentation option for patients with LLD (Belvederi Murri et al., 2015). In addition, a meta-analysis by Bray et al. (2021) showed that exercise increased brain network functional connectivity in older adults, resulting in improved cognitive function. Similar conclusions were drawn in a randomized controlled study by Erickson et al. (2011), in which exercise training increased the volume of hippocampal structures and the concentration of brain-derived neurotrophic factor and was beneficial for memory enhancement in older adults. Thus, physical activity may improve depression and cognitive function in patients with LLD by repairing “disconnected” brain network connections and upregulating neurotrophic factors. Moreover, exercise promotes blood circulation in the brain and protects the cardiovascular and cerebral blood vessels, which may be beneficial for patients with LLD and vascular risk factors.

There are many imaging studies of patients with LLD but no consistent findings regarding the relationship between neurobiological changes and symptoms. Cerebrovascular lesions may induce WMH and white matter microstructural changes, which may lead to dysfunction between and within brain networks. However, longitudinal studies with large samples combined with neuroimaging are needed to further explore the pathophysiology and characteristics of brain network dysfunction in patients with LLD, and we predict that this will be one of the future hotspots in the field.

#### LLD and vascular disease

Numerous studies have shown that a variety of vascular pathologic mechanisms (especially cardiovascular disease, white matter lesions, hypertension, and abnormal impairments such as inflammation and amyloid deposition) are associated with LLD (Petersson et al., 2014; van Sloten et al., 2015; van Agtmaal et al., 2017). A meta-analysis showed that suffering from LLD was associated with an increased risk of cardiovascular disease mortality of more than 30% (Wei et al., 2019). Alexopoulos et al. (1997) first proposed the “vascular depression hypothesis,” which suggests that cerebrovascular diseases (particularly subcortical microvascular and white matter lesions) may damage the structure and function of the frontal cortex, making the onset and development of depressive symptoms more likely in older adults.

A study by Geraets et al. (2021) explored the association between cerebral small vessel disease (CSVD), general brain atrophy markers, LLD, and cognitive dysfunction and found that a larger volume of WMH due to CSVD was associated with MDD in individuals aged > 60 years, whereas general brain atrophy markers were not associated with the incidence of MDD. Another meta-analysis of older adults showed that both peripheral vascular and cerebral microvascular dysfunction were associated with a high prevalence of LLD (van Agtmaal et al., 2017). The results of these studies provide evidence for the “vascular depression hypothesis.” A recent population-based study showed an association between white matter microstructural integrity and longitudinal progression of depression (Shen et al., 2019). Lesions in white matter structures may be associated with increased vulnerability to cognitive function in old age, as well as LLD. Larger WMH volumes are associated with decreased executive function and memory loss in patients with LLD (Oberlin et al., 2022). Cognitive impairment is an important part of LLD; the higher the severity of depression, the greater the risk of cognitive impairment (Jang et al., 2021). Diniz et al. (2013) screened population-based prospective studies and found that depression was associated with an increased risk of all-cause dementia and an increased risk of AD and VD. Interestingly, this study also found that the risk of developing VD was significantly higher in patients with LLD than in those with AD. Therefore, clinical studies with larger sample sizes are necessary to validate the effects of depression prevention on the risk of cognitive impairment and dementia in older adults. LLD is also strongly associated with a higher burden of cardiovascular disease (Sheline et al., 2010; Choi et al., 2014). Wei et al. (2022) used the National Health and Nutrition Examination Survey database to explore the relationship between depression/depressive symptoms and cardiovascular mortality using multivariate proportional risk modeling. They included 8,082 participants in their analysis and found that LLD and its symptoms were associated with increased cardiovascular mortality (Wei et al., 2022). Additionally, genome-wide analyses supported the link between LLD and the risk of vascular dysfunction (Marshe et al., 2021).

Cardiovascular and cerebrovascular diseases may not only change the structure of key areas of the brain and disrupt the structural integrity of the vasculature but also damage neural circuits related to mood and cognition, which can lead to the development of LLD (Vyas and Okereke, 2020). Prevention of vascular risk factors has the potential to reduce the incidence of LLD and the risk of dementia. Diniz et al. (2013) found an increased risk of VD in patients with LLD, which provides additional evidence of a bidirectional relationship between vascular disease and LLD and suggests that the incidence of dementia (especially VD and AD) among older adults can be reduced by preventing depressive disorders. In the future, clinical studies with large sample sizes are needed to support this hypothesis. Therefore, we predict that the relationship between LLD and VD will be a research hotspot.

#### Treatment of LLD

The treatment of LLD is a challenging endeavor. Due to frailty and medical comorbidities, patients’ complaints of depressive symptoms are often masked, leading to a low rate of disease detection. However, some patients are unable to tolerate or adhere to pharmacological interventions and are unable to receive adequate treatment; therefore, they have a worse prognosis than younger patients with depression. A meta-analysis noted that only 4 to 37% of elderly patients with depression received a medication, and a 2-year follow-up of these patients showed that 33% were in remission and 21% died (Lenze et al., 2015). Some studies have shown that LLD is associated with higher rates of physical illness, suicide, and increased mortality from all causes (Bruce and Leaf, 1989; Penninx et al., 1999; Schulz et al., 2000, 2002); however, more than 40 percent of patients with LLD do not receive adequate treatment (Wang et al., 2005; Kok and Reynolds, 2017). Therefore, the timely identification and diagnosis of patients with LLD, allowing them to receive timely and adequate treatment, are current clinical challenges.

Currently, medication combined with psychotherapy is the first-line treatment for patients with LLD. Currently, the main first-line drugs for the treatment of depression are selective serotonin reuptake inhibitor (SSRI), 5-hydroxytryptamine and norepinephrine reuptake inhibitor (SNRI), norepinephrine-ergic and specific 5-hydroxytryptamine-ergic antidepressant (NaSSA), norepinephrine and dopamine reuptake inhibitor (NDRI), while the second-line drugs contain vortioxetine, agomelatine, tricyclic antidepressant and monoamine oxidase inhibitor. However, as people age, their pharmacokinetics change accordingly, resulting in decreased absorption and excretion rates, altered bioavailability, increased serum concentrations of drugs and metabolites, and significantly lower response rates to antidepressants than in younger patients with MDD (Tedeschini et al., 2011). Osteoporosis, hyponatremia, and 5-hydroxytryptamine (5-HT) syndrome, which are rarely seen as side effects of antidepressants in adult patients with depression, are more often seen in the elderly population (Topiwala et al., 2014). In addition, due to medical comorbidities, patients with LLD often receive complex polypharmacy, which increases the risk of drug interactions and multiple adverse effects (Waern et al., 2002). Currently, evidence-based data in the elderly population show a significantly greater effect of antidepressants than placebo in patients with LLD (Nelson et al., 2013), but this advantage may not apply to patients ≥ 65 years (Mallery et al., 2019) but rather to the subgroup of patients aged 55–65 years (Tedeschini et al., 2011). It is hypothesized that the low response rate to antidepressants may be related to the greater frailty of older patients, implying that the evidence-based data do not favor a broad range of antidepressants for patients with LLD. However, most of these studies did not have objective evidence (psychometric or imaging) to rule out the presence of dementia in the patients, so it cannot be assumed that those results are due to the frailty of patients with LLD and that dementia itself can aggravate frailty. Nonetheless, several expert consensuses still recommend the active use of antidepressants in patients with LLD, as pharmacotherapy can reduce suicide rates to some extent (Alexopoulos, 2011; Mulsant et al., 2014). Guidelines for the treatment of depression in both the United States and Canada have prioritized antidepressants in combination with psychotherapy for the treatment of unipolar non-psychotic depression. They recommended selective 5-HT reuptake inhibitors or venlafaxine as first-line treatments for LLD because these drugs have fewer adverse effects and a lower likelihood of drug–drug interactions than other antidepressants (Alexopoulos et al., 2001; Canadian Coalition for Seniors’ Mental Health, 2006).

In addition to antidepressants, atypical antipsychotics and lithium have been shown to be effective in treating depressive symptoms in patients with LLD, and both have been approved as intensive treatments for patients with MDD. A randomized controlled trial by Katila et al. (2013) explored the use of quetiapine in patients with LLD and found that quetiapine monotherapy was effective in terms of depression scores and remission rates compared with placebo. Wilkinson et al. (2002) divided LLD patients (*N* = 50) who had recovered on antidepressants into two groups randomized to additional maintenance treatment with low-dose lithium and placebo, and followed the patients over a two-year period for data on relapse. The results showed that none of the patients with LLD who took additional lithium relapsed after six months, and four of the patients who took only antidepressants relapsed. Two years later, the patients taking additional lithium had significantly lower relapse rates than those taking antidepressants alone (4 vs. 33%) (Wilkinson et al., 2002). This suggests that long-term low-dose lithium reduces the risk of recurrence in patients with LLD, which shows a positive role for lithium in patients with LLD but needs to be validated by studies with larger samples. Simultaneously, we need to be aware of the occurrence of adverse events associated with lithium, especially neurotoxicity and nephrotoxicity. Physical therapy is an effective treatment for LLD. Studies have shown that electroconvulsive therapy not only relieves depression (Dong et al., 2018) but also reduces suicidal ideation (Kellner et al., 2005; Patel et al., 2006). Other studies confirmed the beneficial effects of transcranial magnetic stimulation in patients with LLD (Trevizol et al., 2019).

Research on the efficacy of psychotherapy on LLD has been increasing in recent years. Since the elderly are in a period of social role change, they cannot deal with problems such as social and interpersonal relationships well, and various factors increase the psychological pressure of the elderly, so psychotherapy has an important value in the treatment of depression in the elderly and it is safer (Weissman and Klerman, 2018). Huang et al. (2015) conducted a systematic review and meta-analysis of 27 randomized controlled psychotherapy trials for LLD, which showed that psychotherapy is effective for depression in older adults. One study showed that cognitive behavioral therapy significantly improved patients’ depressive symptoms compared to the control group (Yuan et al., 2020). More importantly, elderly patients with depression benefit more from cognitive-behavioral therapy combined with medication than from medication or psychotherapy alone (Cuijpers et al., 2012). In addition, a recent study on interpersonal psychotherapy points to the influence of interpersonal factors in triggering and maintaining LLD symptoms. Their findings suggest that a relationship-focused intervention in the progression of LLD patients is beneficial, even for those with mild cognitive impairment (Xu and Koszycki, 2020). The effects of physical activity on LLD, besides medication, psychotherapy, and physical therapy, have recently attracted the attention of researchers. Bigarella et al. (2022) conducted a comprehensive review of multiple meta-analyses aimed at assessing the effects of exercise interventions on LLD. Their study found that exercise had a moderate improvement in depressive symptoms in patients with LLD, with aerobic exercise appearing to have a more pronounced effect (Bigarella et al., 2022).

Although there have been numerous studies on treatment options for patients with LLD, clinical trials have typically excluded this population owing to their poorer somatic conditions and more numerous medication side effects (Mottram et al., 2006; Mallery et al., 2019). Many studies on antidepressants in older populations lack clinical data, and the safety of antidepressant use in older adults with comorbid chronic physical illnesses remains unsupported. Little is known about how antidepressants are chosen for LLD patients with cognitive impairment, and whether these drugs further affect cognitive function. Additionally, nonpharmacologic treatments have not been well explored in patients with LLD. Therefore, we believe that the antidepressant treatment of LLD remains a hotspot for future research.

## Limitation

This study has some limitations. First, it is possible that some of the articles that were accepted in 2023 have not yet appeared in print and that the number of citations for recent publications is low; therefore, it is possible that we underestimated their significance. Second, we only searched for literature in English and did not include publications in other languages in the analysis; although the number of non-English publications in this field is small, it is still possible that their contributions were missed.

## Conclusion

Late-life depression has attracted increasing attention from researchers, with more publications each year. However, many unanswered questions remain, such as the relationship between LLD and cognitive impairment and dementia, or the impact of vascular factors and brain network dysfunction on LLD. In addition, the development of more effective treatment approaches for patients with LLD and efficacy-enhancing therapies is one of the current challenges in clinical practice. The results of this study predict future research hotspots and help researchers identify suitable research partners and journals.

## Data availability statement

The raw data supporting the conclusions of this article will be made available by the authors, without undue reservation.

## Author contributions

RD: Data curation, Software, Visualization, Writing – original draft, Writing – review & editing. KY: Investigation, Methodology, Project administration, Writing – original draft. WL: Funding acquisition, Investigation, Methodology, Software, Writing – review & editing. ZW: Funding acquisition, Visualization, Writing – review & editing. HC: Formal analysis, Funding acquisition, Investigation, Methodology, Visualization, Writing – review & editing.
